# Formation and structural characterization of a potassium amidino­guanidinate

**DOI:** 10.1107/S2056989018015980

**Published:** 2018-11-16

**Authors:** Volker Lorenz, Phil Liebing, Liane Hilfert, Sabine Busse, Frank T. Edelmann

**Affiliations:** a Chemisches Institut der Otto-von-Guericke-Universität Magdeburg, Universitätsplatz 2, 39106 Magdeburg, Germany

**Keywords:** crystal structure, amidinate ligands, guanidinate ligands, amidino­guanidinate, potassium, π-coordination

## Abstract

The first potassium amidino­guanidinate complex, *catena*-poly[[bis­(μ-1-amidinato-*N*,*N*′,*N*′′,*N*′′′-tetra­iso­propyl­guanidinato-κ^5^ *N* ^1^:*N* ^1^,*N* ^2^:*N* ^2^,*N* ^4^)dipotassium]-μ-1,2-di­meth­oxy­ethane-κ^2^ *O*:*O*′], [K_2_(C_14_H_32_N_4_)_2_(C_4_H_10_O_2_)]_*n*_ or [{^*i*^PrN= CHN(^*i*^Pr)N(N^*i*^Pr)_2_K}_2_(*μ*-DME)]_*n*_ where DME is 1,2-di­meth­oxy­ethane, has been synthesized and structurally characterized. The title compound was isolated in 76% yield from a reaction of *N*,*N*′-diiso­propyl­carbodi­imide with potassium hydride in DME. The single-crystal X-ray structure determination of the title compound revealed a polymeric chain structure comprising cage-like dimeric units, with the amidino­guanidinate ligand displaying a mixed σ-/π-coordination mode.

## Abstract 2

In the polymeric potassium complex [{^*i*^PrN= CHN(^*i*^Pr)N(N^*i*^Pr)_2_K}_2_(*μ*-DME)]_*n*_, the amidino­guanidinate ligand adopts an unusual mixed σ-/π-coordination mode.

## Chemical context   

Hetero­allylic *N*,*N*′-chelating donor ligands such as amidinate anions [*R*C(N*R*)_2_]^−^ and guanidinate anions [*R*_2_NC(N*R*)_2_]^−^ are of significant importance in various fields of organometallic and coordination chemistry. It is generally accepted that both types of *N*,*N*′-chelating ligands can be regarded as ‘steric cyclo­penta­dienyl equivalents’ (Bailey & Pace, 2001 ▸; Collins, 2011 ▸; Edelmann, 2013 ▸). Over the past three decades, amidinato and guanidinato complexes have been synthesized for nearly every metallic element in the Periodic Table ranging from lithium to the *f*-block elements (Edelmann, 2009 ▸, 2012 ▸, 2013 ▸; Trifonov, 2010 ▸). Important applications of amidinate and guanidinate ligands include the stabilization of unusually low oxidation states (*e.g*. Mg^I^ and Fe^I^) as well as the design of highly active homogeneous catalysts (Collins, 2011 ▸; Edelmann, 2013 ▸; Chen *et al.*, 2018 ▸). Metal amidinate and guanidinate complexes bearing small aliphatic substituents have also been established as ALD (= atomic layer deposition) and MOCVD (= metal–organic chemical vapor deposition) precursors for the deposition of thin films of metals, metal oxides, metal nitrides *etc.* (Devi, 2013 ▸). Formally, amidinate anions are nitro­gen analogues of carboxyl­ate anions, while guanidinates are related in the same way to carbamate anions. However, in contrast to the carboxyl­ates and carbamates, the steric properties of amidinates and guanidinates can be tuned over a wide range by employing different substituents at the outer nitro­gen atoms as well as at the central carbon atom of the chelating NCN unit. The most important starting materials in this area are lithium amidinates, which are normally prepared in a straightforward manner by the addition of lithium alkyls to *N*,*N*′-diorganocarbodi­imides in a 1:1 molar ratio. Lithium guanidinates are formed in the same manner by adding lithium-*N,N*-di­alkyl­amides to *N*,*N*′-diorganocarbodi­imides (Stalke *et al.*, 1992 ▸; Aharonovich *et al.*, 2008 ▸; Chlupatý *et al.*, 2011 ▸; Nevoralová *et al.*, 2013 ▸; Hong *et al.*, 2013 ▸). All of these reactions are generally quite straightforward and afford the desired products in high yields. Less investigated are amidin­ate salts of the heavier alkali metals sodium and potassium (Cole *et al.*, 2003 ▸; Cole & Junk, 2003 ▸; Junk & Cole, 2007 ▸; Yao *et al.*, 2009 ▸; Dröse *et al.*, 2010 ▸, Chen *et al.*, 2018 ▸).
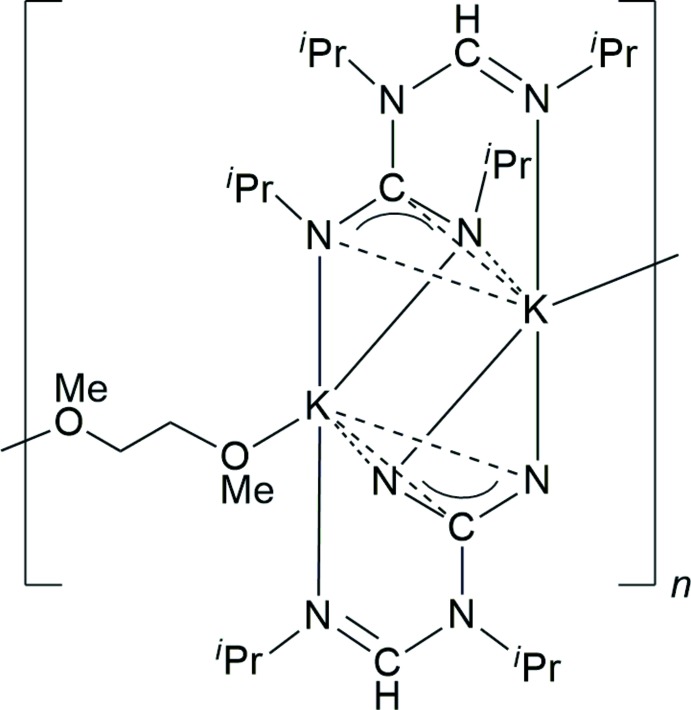


We recently reported in this journal that, under certain conditions, seemingly straightforward reactions of lithium alkyls with *N*,*N*′-diorganocarbodi­imides can take a different course, leading to lithium salts of dimerized amidinates ligands (‘amidino­guanidinates’) (Sroor *et al.*, 2016 ▸). These could even become the major reaction products when the *N*,*N*′-diorg­ano­carbodi­imides are used in a twofold molar excess. The first complexes comprising amidino­guanidinate ligands included the lithium precursors Li[^*n*^BuC(=N*R*)(N*R*)C(N*R*)_2_] [*R* = ^*i*^Pr, Cy (= cyclo­hex­yl)] and the holmium(III) complex [^*n*^BuC(=NCy)(NCy)C(NCy)_2_]Ho[^*n*^BuC(NCy)_2_](μ-Cl)_2_Li(THF)_2_ (Sroor *et al.*, 2016 ▸). In this contribution, we report the synthesis and structural characterization of the first potassium amidino­guanidinate derivative, polymeric *catena*-poly[[bis­(μ-1-amidinato-*N*,*N*′,*N*′′,*N*′′′-tetra­iso­propyl­guanidinato-κ^5^ *N* ^1^:*N* ^1^,*N* ^2^:*N* ^2^,*N* ^4^)dipotassium]-μ-1,2-di­meth­oxy­ethane-κ^2^ *O*:*O*′] [{^*i*^PrN=CHN(^*i*^Pr)N(N^*i*^Pr)_2_K}_2_(*μ*-DME)]_*n*_.

**Figure 1:**
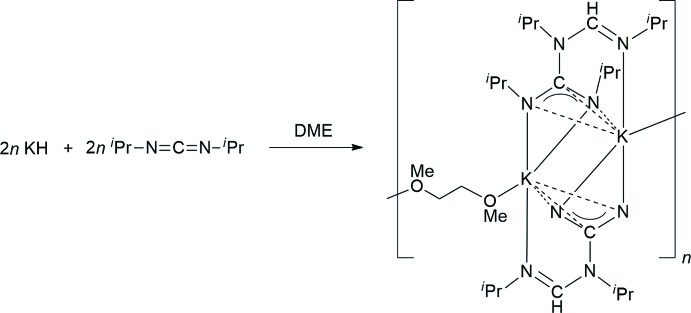
Formation of the title compound by reaction of potassium hydride with *N*,*N*′-diiso­propyl­carbodi­imide in DME.

As illustrated in Fig. 1 ▸, the title compound was formed when *N*,*N*′-diiso­propyl­carbodi­imide was added to a suspension of potassium hydride in 1,2-di­meth­oxy­ethane (DME). With the attempt to prepare the corresponding amidinate K[HC(N^*i*^Pr)_2_], the reactants were used in a molar ratio 1:1. After filtration and concentration of the filtrate to a small volume, the product crystallized directly at room temperature and could be isolated as colorless, plate-like, moisture-sensitive crystals in 76% yield (calculated after determination of the crystal structure). The compound was characterized through elemental analysis as well as IR, NMR (^1^H and ^13^C) and mass spectra. However, the usual set of analytical and spectroscopic methods did not allow for an unequivocal elucidation of the mol­ecular structure. NMR data clearly indicated the presence of coordinated DME. However, both the ^1^H and ^13^C NMR spectra showed two sets of *iso*-propyl resonances, thereby ruling out the formation of a simple potassium formamidinate salt of the composition ‘(DME)K[HC(N^*i*^Pr)_2_]’. Fortunately, the single crystals obtained directly from the filtered and concentrated reaction solution were suitable for X-ray diffraction analysis. This study confirmed the formation of a new amidino­guanidinate complex through dimerization of *N*,*N*′-diiso­propyl­carbodi­imide in the coordination sphere of potassium.

## Structural commentary   

**Figure 2:**
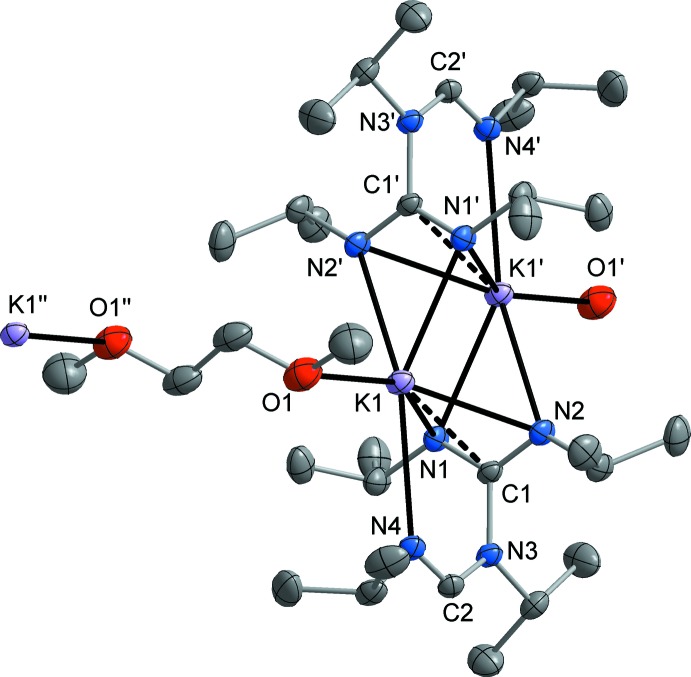
Mol­ecular structure of the title compound in the crystal. Displacement ellipsoids are drawn at the 50% probability level, hydrogen atoms omitted for clarity. Symmetry codes: (′) −*x*, −*y*, 2 − *z*; (′′) −*x*, −1 − *y*, 2 − *z*.

**Figure 3:**
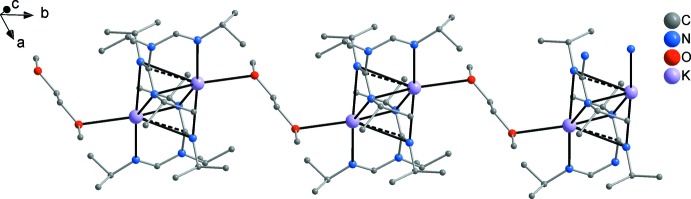
Illustration of the polymeric chain structure of the title compound, extending along the crystallographic *b* axis.

The mol­ecular structure of the title compound consists of centrosymmetric dimeric units, being composed of two potassium atoms and two amidino­guanidinate ligands (Fig. 2 ▸). The guanidinate unit is attached to potassium in an *N*,*N*′-chelating mode, with the K atom in the N_3_C plane of the guanidinate. The same guanidinate moiety is linked to the symmetry-equivalent K atom in an η^3^-di­aza­allyl mode, *i.e*. the metal atom is situated above the N1/C1/N2 plane. The exposed nitro­gen donor of the amidinate backbone (N4) in the title compound is attached to the metal center in a simple monodentate coordination, with the N atom having a perfectly planar environment (sum of bond angles = 360.0°). This is in agreement with the expected *sp* ^2^ hybridization of atom N4 (*cf*. Scheme[Chem scheme1]). As a result of the μ-bridging coordination of the amidino­guanidinate ligand, the potassium atom is surrounded by a σ-chelating guanidinate group, a π-di­aza­allyl-coordinated guanidinate group, and a single amidinate nitro­gen atom in a T-shaped fashion. A pseudo-square-planar coordination is completed by one oxygen atom of a μ-κ*O*:κ*O′-*coordinated DME ligand. Through this bridging DME coordination, the dimeric units are inter­connected into a one-dimensional coordination polymer (Fig. 3 ▸).

**Figure 4:**
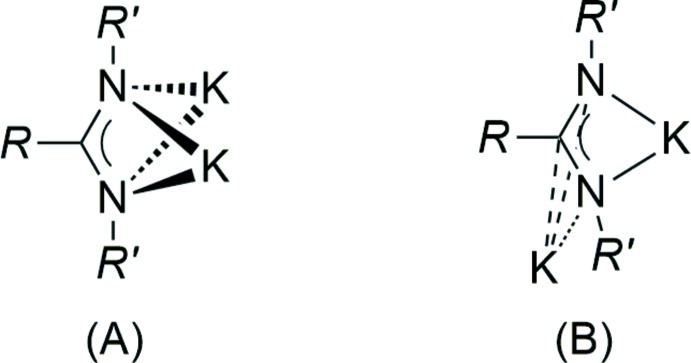
Coordination modes of 1,3-di­aza­allyl-type ligands (= amidinate or guanidinate) observed in potassium complexes: symmetric double-chelating (A), single-chelating and η^3^-coordination of the 1,3-di­aza­allyl π-system (B).

An increased tendency towards π-coordination modes is characteristic for the heavier alkali metals and has frequently been observed in other complexes with nitro­gen ligands (*e.g*. von Bülow *et al.*, 2004 ▸; Liebing & Merzweiler, 2015 ▸). However, in potassium amidinates and guanidinates, a symmetric double-chelating coordination is usually preferred over coordination modes with a contribution of the π-electron system (Fig. 4 ▸) (Giesbrecht *et al.*, 1999 ▸; Benndorf *et al.*, 2011 ▸). A similar mixed σ-/π-coordination to that in the title compound has been recently observed by us in a potassium di­thio­carbamate (Liebing, 2017 ▸).

The K—N bond lengths to the σ-bonded guanidinate group are 2.793 (2) Å (N1) and 2.814 (2) Å (N2), while the bond to the single amidinate nitro­gen donor (N4) is considerably longer at 2.939 (2) Å. All these values are within the range usually observed for K—N bonds (crystal structures deposited in the CSD; Groom *et al.*, 2016 ▸). The K—N distances to the π-coordinated guanidinate group are 2.882 (2) Å (N1) and 2.979 (2) Å (N2), and the corresponding K—C1 separation was determined to be 2.967 (2) Å. The latter value is considerably smaller than in a structurally related potassium di­thio­carbamate [K—C 3.150 (2) Å; Liebing, 2017 ▸].

## Supra­molecular features   

The crystal structure of the title compound does not display any specific inter­actions between the polymeric chains. The closest inter­chain contact is 3.632 (3) Å (C5⋯C14) between the methyl carbon atoms of isopropyl groups.

## Database survey   

For a review article on related alkali metal bis­(ar­yl)formamidinates, see: Junk & Cole (2007 ▸). For other structurally characterized alkali metal amidinates and guanidinates, see: Giesbrecht *et al.* (1999 ▸), Stalke *et al.* (1992 ▸), Cole *et al.* (2003 ▸), Aharonovich *et al.* (2008 ▸), Chlupatý *et al.* (2011 ▸), Cole & Junk (2003 ▸), Junk & Cole (2007 ▸), Benndorf *et al.* (2011 ▸), Nevoralová *et al.* (2013 ▸) and Hong *et al.* (2013 ▸).

## Synthesis and crystallization   

**General Procedures:** The reaction was carried out under an inert atmosphere of dry argon employing standard Schlenk and glove-box techniques. The solvent di­meth­oxy­ethane (DME) was distilled from sodium/benzo­phenone under nitro­gen atmosphere prior to use. All glassware was oven-dried at 393 K for at least 24 h, assembled while hot, and cooled under high vacuum prior to use. The starting material *N*,*N*′-diiso­propyl­carbodi­imide was obtained from Sigma–Aldrich and used as received. Commercially available potassium hydride was freed from protecting paraffin oil by thoroughly washing with *n*-pentane and stored in a glove-box. The ^1^H and ^13^C NMR spectra were recorded in solutions on a Bruker Biospin AVIII 400 MHz spectrometer at 298 K. Chemical shifts are referenced to tetra­methyl­silane. The IR spectrum was measured with a Bruker Optics VERTEX 70v spectrometer, and the electron impact mass spectrum was recorded using a MAT95 spectrometer with an ionization energy of 70 eV. Microanalysis of the title compound was performed using a ‘vario EL cube’ apparatus from Elementar Analysensysteme GmbH. The melting/decomposition point was measured on a Büchi Melting Point B-540 apparatus.

**Synthesis of [{** ***^i^*** **PrN**=**CHN(** ***^i^*** **Pr)N(N** ***^i^*** **Pr)_2_K}_2_(** ***μ*** **-DME)]** ***_n_*** **:** 1.6 mL (1.26 g, 10.0 mmol) of *N*,*N*′-diiso­propyl­carbodi­imide were added to a stirred suspension of 0.41 g (10 mmol) of KH in 50 ml of DME. The reaction mixture was stirred for two days and refluxed for an additional 2 h. After cooling to room temperature, all insoluble solid parts were filtered off and the volume of the resulting clear solution was reduced to *ca* 25 ml. After three days at room temperature, the title compound crystallized as colorless, plate-like crystals suitable for single-crystal X-ray diffraction. Yield: 1.3 g (76%). M.p. 378 K (dec.). C_32_H_68_K_2_N_8_O_2_ (*M* = 675.15 g mol^−1^): calculated C 56.93, H 10.15, N 16.60; found: C 56.81, H 10.24, N 16.33%. **IR** (ATR): *ν* = 2952 *m*, 2858 *m*, 2824 *w*, 1626 *m*, 1538 *s*, 1465 *m*, 1453 *m*, 1383 *m*, 1369 *m*, 1358 *m*, 1343 *m*, 1318 *m*, 1298 *m*, 1196 *m*, 1162 *m*, 1125 *m*, 1111 *m* 1048 *w*, 993 *m*, 955 *w*, 946 *w*, 858 *w*, 815 *w*, 674 *w*, 575 *w*, 516 *w*, *442 m* 373 *w*, 338 *m*, 295 *w*, 262 *m* cm^−1^. **^1^H NMR** (400.1 MHz, THF-*d*_8_, 293 K): *δ* = 7.90 (*s*, 2H, N—C*H*=N), 3.47 [*sept*, 4H, C*H*(CH_3_)_2_], 3.43 (*s*, 8H, DME), 3.27 (*s*, 12H, DME), 3.01 [*sept*, 4H, C*H*(CH_3_)_2_], 1.15 [*d*, 24H, CH(C*H*_3_)_2_], 0.94 [*d*, 24H, CH(C*H*_3_)_2_] ppm. **^13^C NMR** (100.6 MHz, THF-*d*_8_, 293 K): *δ* = 166.0 (N—*C*H=N), 150.0 (N—*C*N—N), 72.6 (DME), 58.9 (DME), 55.5 [*C*H(CH_3_)_2_], 49.4 [*C*H(CH_3_)_2_], 28.2 [CH(*C*H_3_)_2_], 25.0 [CH(*C*H_3_)_2_] ppm. **MS** (EI, 70 eV): *m*/*z* = 254 (5) [C_14_H_30_N_4_]^+^, 211 (30) [C_14_H_30_N_4_ − ^*i*^Pr]^+^, 184 (32), 170 (38), 144 (82), 129 (100).

## Refinement   

Crystal data, data collection and structure refinement details are summarized in Table 1 ▸. H atoms attached to C atoms were fixed geometrically and refined using a riding model. CH_3_ groups were allowed to rotate freely around the C—C vector, and the corresponding C—H distances were constrained to 0.98 Å. C—H distances within CH_2_ groups were constrained to 0.99 Å, C—H distances within the ^*i*^Pr CH groups to 1.00 Å, and the C—H distance within the amidinate group (*i.e.* at C2) to 0.95 Å. The *U*_iso_(H) values were set at 1.5*U*_eq_(C) for methyl groups and at 1.2*U*_eq_(C) in all other cases. The reflections (001) and (010) disagreed strongly with the structural model and were therefore omitted from the refinement.

## Supplementary Material

Crystal structure: contains datablock(s) I. DOI: 10.1107/S2056989018015980/zl2742sup1.cif

Structure factors: contains datablock(s) I. DOI: 10.1107/S2056989018015980/zl2742Isup2.hkl

CCDC reference: 1862025

Additional supporting information:  crystallographic information; 3D view; checkCIF report

## Figures and Tables

**Table 1:** Experimental details

Crystal data	
Chemical formula	[K_2_(C_14_H_32_N_4_)_2_(C_4_H_10_O_2_)]
*M*_r_	337.57
Crystal system, space group	Triclinic, *P* 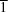
Temperature (K)	153
*a*, *b*, *c* (Å)	10.3207 (6), 10.5311 (6), 11.6703 (7)
α, β, γ (°)	71.605 (4), 64.168 (4), 63.516 (4)
*V* (Å^3^)	1010.23 (11)
*Z*	2
Radiation type	Mo *K*α
μ (mm^−1^)	0.27
Crystal size (mm)	0.46 × 0.37 × 0.16

Data collection	
Diffractometer	STOE IPDS 2T
No. of measured, independent and observed [*I* > 2σ(*I*)] reflections	9148, 3941, 3368
*R*_int_	0.104
(sin θ/λ)_max_ (Å^−1^)	0.617

Refinement	
*R*[*F* ^2^ > 2σ(*F* ^2^)], *wR*(*F* ^2^), *S*	0.051, 0.139, 1.03
No. of reflections	3941
No. of parameters	208
H-atom treatment	H-atom parameters constrained
Δρ_max_, Δρ_min_ (e Å^−3^)	0.41, −0.68

Computer programs: *X-AREA* and *X-RED* (Stoe & Cie, 2002 ▸), *SIR97* (Altomare *et al.*, 1999 ▸), *SHELXL2018/3* (Sheldrick, 2015 ▸), *DIAMOND* (Brandenburg, 1999 ▸) and *publCIF* (Westrip, 2010 ▸).

## supplementary crystallographic information

### Crystal data

**Table:**

[K_2_(C_14_H_32_N_4_)_2_(C_4_H_10_O_2_)]	*Z* = 2
*M**_r_* = 337.57	*F*(000) = 370
Triclinic, *P*1	*D*_x_ = 1.110 Mg m^−^^3^
*a* = 10.3207 (6) Å	Mo *K*α radiation, λ = 0.71073 Å
*b* = 10.5311 (6) Å	Cell parameters from 12337 reflections
*c* = 11.6703 (7) Å	θ = 2.0–29.2°
α = 71.605 (4)°	µ = 0.27 mm^−^^1^
β = 64.168 (4)°	*T* = 153 K
γ = 63.516 (4)°	Plate, colorless
*V* = 1010.23 (11) Å^3^	0.46 × 0.37 × 0.16 mm

### Data collection

**Table:**

STOE IPDS 2T diffractometer	3368 reflections with *I* > 2σ(*I*)
Radiation source: fine-focus sealed tube	*R*_int_ = 0.104
Detector resolution: 6.67 pixels mm^-1^	θ_max_ = 26.0°, θ_min_ = 2.4°
area detector scans	*h* = −12→12
9148 measured reflections	*k* = −12→12
3941 independent reflections	*l* = −14→14

### Refinement

**Table:**

Refinement on *F*^2^	Primary atom site location: heavy-atom method
Least-squares matrix: full	Secondary atom site location: difference Fourier map
*R*[*F*^2^ > 2σ(*F*^2^)] = 0.051	Hydrogen site location: inferred from neighbouring sites
*wR*(*F*^2^) = 0.139	H-atom parameters constrained
*S* = 1.03	*w* = 1/[σ^2^(*F*_o_^2^) + (0.0873*P*)^2^] where *P* = (*F*_o_^2^ + 2*F*_c_^2^)/3
3941 reflections	(Δ/σ)_max_ = 0.001
208 parameters	Δρ_max_ = 0.41 e Å^−^^3^
0 restraints	Δρ_min_ = −0.68 e Å^−^^3^

### Special details

**Table:**

Geometry. All esds (except the esd in the dihedral angle between two l.s. planes) are estimated using the full covariance matrix. The cell esds are taken into account individually in the estimation of esds in distances, angles and torsion angles; correlations between esds in cell parameters are only used when they are defined by crystal symmetry. An approximate (isotropic) treatment of cell esds is used for estimating esds involving l.s. planes.

### Fractional atomic coordinates and isotropic or equivalent isotropic displacement parameters (Å^2^)

**Table:**

	*x*	*y*	*z*	*U*_iso_*/*U*_eq_	
C1	0.1180 (2)	0.10787 (17)	0.76592 (16)	0.0246 (4)	
C2	0.2802 (2)	−0.03149 (19)	0.59010 (17)	0.0258 (4)	
H1	0.343661	−0.037395	0.502260	0.031*	
C3	−0.1057 (2)	0.1371 (2)	0.72593 (19)	0.0334 (4)	
H2	−0.033813	0.142578	0.634615	0.040*	
C4	−0.1524 (3)	0.0079 (2)	0.7597 (2)	0.0423 (5)	
H4	−0.207873	0.020053	0.704601	0.051*	
H3	−0.219625	0.000043	0.850132	0.051*	
H5	−0.059844	−0.079402	0.745993	0.051*	
C5	−0.2492 (3)	0.2737 (3)	0.7413 (3)	0.0484 (6)	
H7	−0.306745	0.278110	0.691062	0.073*	
H8	−0.218236	0.357464	0.710293	0.073*	
H6	−0.314695	0.273254	0.832358	0.073*	
C6	0.3490 (2)	0.0683 (2)	0.7999 (2)	0.0333 (4)	
H9	0.389908	0.086282	0.703653	0.040*	
C7	0.4370 (3)	−0.0860 (3)	0.8468 (2)	0.0436 (5)	
H10	0.545801	−0.099728	0.821237	0.065*	
H12	0.429255	−0.151624	0.808497	0.065*	
H11	0.392692	−0.105796	0.940845	0.065*	
C8	0.3718 (3)	0.1705 (3)	0.8520 (3)	0.0480 (6)	
H14	0.481983	0.153889	0.821431	0.072*	
H15	0.331857	0.153064	0.946321	0.072*	
H13	0.315981	0.269944	0.821474	0.072*	
C9	0.2355 (2)	0.22472 (19)	0.53339 (18)	0.0308 (4)	
H16	0.349595	0.201466	0.493239	0.037*	
C10	0.1732 (3)	0.2586 (2)	0.4266 (2)	0.0446 (5)	
H17	0.205883	0.333363	0.360018	0.067*	
H19	0.060151	0.292378	0.462140	0.067*	
H18	0.213228	0.171778	0.388692	0.067*	
C11	0.1659 (4)	0.3553 (2)	0.5973 (2)	0.0507 (6)	
H21	0.182135	0.437472	0.531850	0.076*	
H22	0.215296	0.337369	0.658908	0.076*	
H20	0.054980	0.376198	0.642701	0.076*	
C12	0.3481 (2)	−0.2763 (2)	0.60265 (19)	0.0340 (4)	
H23	0.414440	−0.253644	0.512021	0.041*	
C13	0.2278 (3)	−0.3213 (3)	0.6014 (3)	0.0536 (6)	
H25	0.279619	−0.407185	0.559670	0.080*	
H26	0.164617	−0.243316	0.553660	0.080*	
H24	0.161667	−0.342468	0.690020	0.080*	
C14	0.4483 (3)	−0.3951 (2)	0.6748 (2)	0.0494 (6)	
H27	0.502364	−0.480376	0.632053	0.074*	
H29	0.383544	−0.418301	0.763433	0.074*	
H28	0.523942	−0.363514	0.675633	0.074*	
C15	0.2643 (4)	−0.5193 (4)	1.0541 (3)	0.0671 (8)	
H32	0.344120	−0.476641	1.009850	0.101*	
H31	0.312830	−0.623859	1.058811	0.101*	
H30	0.206810	−0.494569	1.141425	0.101*	
C16	0.0442 (3)	−0.5234 (2)	1.0445 (2)	0.0473 (6)	
H34	−0.025475	−0.488497	1.127880	0.057*	
H33	0.089693	−0.629485	1.060744	0.057*	
N1	−0.02933 (18)	0.12443 (17)	0.81012 (15)	0.0280 (3)	
N2	0.18526 (17)	0.09405 (17)	0.84450 (15)	0.0282 (3)	
N3	0.20814 (18)	0.09943 (16)	0.62832 (15)	0.0265 (3)	
N4	0.26831 (18)	−0.14760 (16)	0.66462 (15)	0.0278 (3)	
O1	0.1619 (2)	−0.46565 (17)	0.98535 (16)	0.0478 (4)	
K1	0.08968 (5)	−0.15986 (4)	0.94197 (4)	0.02868 (15)	

### Atomic displacement parameters (Å^2^)

**Table:**

	*U*^11^	*U*^22^	*U*^33^	*U*^12^	*U*^13^	*U*^23^
C1	0.0225 (8)	0.0230 (8)	0.0240 (8)	−0.0074 (7)	−0.0036 (7)	−0.0060 (6)
C2	0.0208 (8)	0.0280 (9)	0.0252 (9)	−0.0077 (7)	−0.0031 (7)	−0.0083 (7)
C3	0.0260 (9)	0.0427 (11)	0.0315 (10)	−0.0131 (8)	−0.0093 (8)	−0.0056 (8)
C4	0.0296 (10)	0.0456 (12)	0.0566 (14)	−0.0113 (9)	−0.0133 (10)	−0.0196 (10)
C5	0.0423 (13)	0.0415 (12)	0.0665 (16)	−0.0096 (10)	−0.0336 (12)	−0.0016 (10)
C6	0.0193 (9)	0.0454 (11)	0.0331 (10)	−0.0124 (8)	−0.0045 (8)	−0.0089 (8)
C7	0.0270 (10)	0.0482 (12)	0.0511 (13)	−0.0049 (9)	−0.0141 (10)	−0.0141 (10)
C8	0.0339 (11)	0.0525 (13)	0.0652 (16)	−0.0197 (10)	−0.0167 (11)	−0.0130 (11)
C9	0.0290 (9)	0.0281 (9)	0.0306 (10)	−0.0125 (8)	−0.0064 (8)	−0.0013 (7)
C10	0.0593 (15)	0.0372 (11)	0.0388 (12)	−0.0190 (11)	−0.0221 (11)	0.0021 (9)
C11	0.0791 (18)	0.0309 (11)	0.0438 (13)	−0.0248 (12)	−0.0210 (13)	−0.0008 (9)
C12	0.0331 (10)	0.0280 (9)	0.0324 (10)	−0.0101 (8)	0.0000 (8)	−0.0118 (7)
C13	0.0543 (15)	0.0539 (14)	0.0586 (15)	−0.0274 (12)	−0.0031 (13)	−0.0283 (12)
C14	0.0427 (12)	0.0291 (10)	0.0476 (13)	0.0001 (9)	−0.0029 (11)	−0.0080 (9)
C15	0.077 (2)	0.0673 (18)	0.0608 (18)	−0.0303 (16)	−0.0307 (16)	0.0023 (14)
C16	0.0517 (14)	0.0360 (11)	0.0388 (12)	−0.0176 (10)	−0.0021 (11)	−0.0035 (9)
N1	0.0207 (7)	0.0335 (8)	0.0283 (8)	−0.0087 (6)	−0.0071 (6)	−0.0064 (6)
N2	0.0192 (7)	0.0338 (8)	0.0294 (8)	−0.0084 (6)	−0.0057 (6)	−0.0078 (6)
N3	0.0257 (7)	0.0244 (7)	0.0251 (8)	−0.0092 (6)	−0.0038 (6)	−0.0053 (6)
N4	0.0242 (8)	0.0260 (8)	0.0284 (8)	−0.0072 (6)	−0.0035 (7)	−0.0090 (6)
O1	0.0516 (10)	0.0365 (8)	0.0448 (9)	−0.0172 (7)	−0.0098 (8)	−0.0012 (6)
K1	0.0274 (2)	0.0255 (2)	0.0275 (2)	−0.00806 (16)	−0.00473 (17)	−0.00624 (14)

### Geometric parameters (Å, º)

**Table:**

C1—N2	1.313 (2)	C10—H17	0.9800
C1—N1	1.321 (2)	C10—H19	0.9800
C1—N3	1.472 (2)	C10—H18	0.9800
C1—K1	2.9685 (17)	C11—H21	0.9800
C1—K1^i^	3.2060 (18)	C11—H22	0.9800
C2—N4	1.279 (2)	C11—H20	0.9800
C2—N3	1.356 (2)	C12—N4	1.464 (2)
C2—H1	0.9500	C12—C14	1.511 (3)
C3—N1	1.450 (2)	C12—C13	1.520 (3)
C3—C4	1.523 (3)	C12—H23	1.0000
C3—C5	1.525 (3)	C13—H25	0.9800
C3—H2	1.0000	C13—H26	0.9800
C4—K1	3.495 (2)	C13—H24	0.9800
C4—H4	0.9800	C14—H27	0.9800
C4—H3	0.9800	C14—H29	0.9800
C4—H5	0.9800	C14—H28	0.9800
C5—H7	0.9800	C15—O1	1.414 (4)
C5—H8	0.9800	C15—K1	3.483 (3)
C5—H6	0.9800	C15—H32	0.9800
C6—N2	1.454 (2)	C15—H31	0.9800
C6—C7	1.523 (3)	C15—H30	0.9800
C6—C8	1.531 (3)	C16—O1	1.409 (3)
C6—H9	1.0000	C16—C16^ii^	1.502 (5)
C7—H10	0.9800	C16—H34	0.9900
C7—H12	0.9800	C16—H33	0.9900
C7—H11	0.9800	N1—K1^i^	2.7931 (16)
C8—H14	0.9800	N1—K1	2.8809 (16)
C8—H15	0.9800	N2—K1^i^	2.8135 (16)
C8—H13	0.9800	N2—K1	2.9786 (16)
C9—N3	1.478 (2)	N4—K1	2.9394 (16)
C9—C11	1.504 (3)	O1—K1	2.8880 (16)
C9—C10	1.520 (3)	K1—K1^i^	3.4252 (8)
C9—H16	1.0000		
			
N2—C1—N1	120.49 (16)	K1—C15—H32	72.0
N2—C1—N3	120.05 (15)	O1—C15—H31	109.5
N1—C1—N3	119.42 (15)	K1—C15—H31	160.6
N2—C1—K1	77.68 (10)	H32—C15—H31	109.5
N1—C1—K1	73.27 (10)	O1—C15—H30	109.5
N3—C1—K1	118.28 (10)	K1—C15—H30	87.3
N2—C1—K1^i^	60.96 (10)	H32—C15—H30	109.5
N1—C1—K1^i^	60.10 (10)	H31—C15—H30	109.5
N3—C1—K1^i^	174.37 (11)	O1—C16—C16^ii^	108.0 (2)
K1—C1—K1^i^	67.26 (4)	O1—C16—H34	110.1
N4—C2—N3	124.26 (17)	C16^ii^—C16—H34	110.1
N4—C2—H1	117.9	O1—C16—H33	110.1
N3—C2—H1	117.9	C16^ii^—C16—H33	110.1
N1—C3—C4	110.43 (17)	H34—C16—H33	108.4
N1—C3—C5	109.08 (17)	C1—N1—C3	121.64 (16)
C4—C3—C5	109.46 (17)	C1—N1—K1^i^	95.69 (11)
N1—C3—H2	109.3	C3—N1—K1^i^	141.98 (12)
C4—C3—H2	109.3	C1—N1—K1	80.68 (10)
C5—C3—H2	109.3	C3—N1—K1	115.75 (11)
C3—C4—K1	87.31 (11)	K1^i^—N1—K1	74.25 (4)
C3—C4—H4	109.5	C1—N2—C6	121.77 (16)
K1—C4—H4	159.9	C1—N2—K1^i^	94.96 (11)
C3—C4—H3	109.5	C6—N2—K1^i^	142.90 (12)
K1—C4—H3	73.4	C1—N2—K1	76.81 (10)
H4—C4—H3	109.5	C6—N2—K1	117.91 (11)
C3—C4—H5	109.5	K1^i^—N2—K1	72.44 (4)
K1—C4—H5	52.6	C2—N3—C1	118.08 (14)
H4—C4—H5	109.5	C2—N3—C9	118.91 (15)
H3—C4—H5	109.5	C1—N3—C9	122.73 (14)
C3—C5—H7	109.5	C2—N4—C12	115.65 (16)
C3—C5—H8	109.5	C2—N4—K1	123.14 (12)
H7—C5—H8	109.5	C12—N4—K1	121.21 (11)
C3—C5—H6	109.5	C16—O1—C15	112.12 (19)
H7—C5—H6	109.5	C16—O1—K1	121.11 (14)
H8—C5—H6	109.5	C15—O1—K1	102.68 (15)
N2—C6—C7	110.45 (16)	N1^i^—K1—N2^i^	48.14 (4)
N2—C6—C8	109.34 (17)	N1^i^—K1—N1	105.75 (4)
C7—C6—C8	109.17 (18)	N2^i^—K1—N1	88.98 (5)
N2—C6—H9	109.3	N1^i^—K1—O1	98.99 (5)
C7—C6—H9	109.3	N2^i^—K1—O1	97.20 (5)
C8—C6—H9	109.3	N1—K1—O1	151.41 (5)
C6—C7—H10	109.5	N1^i^—K1—N4	152.08 (4)
C6—C7—H12	109.5	N2^i^—K1—N4	153.54 (5)
H10—C7—H12	109.5	N1—K1—N4	70.20 (4)
C6—C7—H11	109.5	O1—K1—N4	94.19 (5)
H10—C7—H11	109.5	N1^i^—K1—C1	107.91 (5)
H12—C7—H11	109.5	N2^i^—K1—C1	109.93 (5)
C6—C8—H14	109.5	N1—K1—C1	26.05 (5)
C6—C8—H15	109.5	O1—K1—C1	150.33 (5)
H14—C8—H15	109.5	N4—K1—C1	56.14 (5)
C6—C8—H13	109.5	N1^i^—K1—N2	87.43 (4)
H14—C8—H13	109.5	N2^i^—K1—N2	107.56 (4)
H15—C8—H13	109.5	N1—K1—N2	45.91 (4)
N3—C9—C11	111.21 (16)	O1—K1—N2	151.32 (5)
N3—C9—C10	112.21 (15)	N4—K1—N2	69.83 (4)
C11—C9—C10	109.47 (18)	C1—K1—N2	25.51 (5)
N3—C9—H16	107.9	N1^i^—K1—C1^i^	24.21 (4)
C11—C9—H16	107.9	N2^i^—K1—C1^i^	24.08 (4)
C10—C9—H16	107.9	N1—K1—C1^i^	99.77 (5)
C9—C10—H17	109.5	O1—K1—C1^i^	96.93 (5)
C9—C10—H19	109.5	N4—K1—C1^i^	168.88 (4)
H17—C10—H19	109.5	C1—K1—C1^i^	112.74 (4)
C9—C10—H18	109.5	N2—K1—C1^i^	99.86 (4)
H17—C10—H18	109.5	N1^i^—K1—K1^i^	54.05 (3)
H19—C10—H18	109.5	N2^i^—K1—K1^i^	56.01 (3)
C9—C11—H21	109.5	N1—K1—K1^i^	51.71 (3)
C9—C11—H22	109.5	O1—K1—K1^i^	149.99 (4)
H21—C11—H22	109.5	N4—K1—K1^i^	115.82 (3)
C9—C11—H20	109.5	C1—K1—K1^i^	59.68 (3)
H21—C11—H20	109.5	N2—K1—K1^i^	51.55 (3)
H22—C11—H20	109.5	C1^i^—K1—K1^i^	53.06 (3)
N4—C12—C14	109.95 (18)	N1^i^—K1—C15	82.81 (6)
N4—C12—C13	108.55 (17)	N2^i^—K1—C15	98.69 (7)
C14—C12—C13	110.70 (19)	N1—K1—C15	171.13 (7)
N4—C12—H23	109.2	O1—K1—C15	23.33 (6)
C14—C12—H23	109.2	N4—K1—C15	101.16 (7)
C13—C12—H23	109.2	C1—K1—C15	149.29 (7)
C12—C13—H25	109.5	N2—K1—C15	134.07 (6)
C12—C13—H26	109.5	C1^i^—K1—C15	89.00 (6)
H25—C13—H26	109.5	K1^i^—K1—C15	136.79 (6)
C12—C13—H24	109.5	N1^i^—K1—C4	128.35 (5)
H25—C13—H24	109.5	N2^i^—K1—C4	85.06 (5)
H26—C13—H24	109.5	N1—K1—C4	43.73 (5)
C12—C14—H27	109.5	O1—K1—C4	108.80 (5)
C12—C14—H29	109.5	N4—K1—C4	68.65 (5)
H27—C14—H29	109.5	C1—K1—C4	63.44 (5)
C12—C14—H28	109.5	N2—K1—C4	87.95 (5)
H27—C14—H28	109.5	C1^i^—K1—C4	107.77 (5)
H29—C14—H28	109.5	K1^i^—K1—C4	84.16 (4)
O1—C15—K1	53.99 (12)	C15—K1—C4	132.11 (6)
O1—C15—H32	109.5		
			
N1—C3—C4—K1	−13.87 (15)	C7—C6—N2—C1	−107.2 (2)
C5—C3—C4—K1	−133.98 (16)	C8—C6—N2—C1	132.6 (2)
N2—C1—N1—C3	−178.76 (16)	C7—C6—N2—K1^i^	81.9 (2)
N3—C1—N1—C3	−1.1 (2)	C8—C6—N2—K1^i^	−38.3 (3)
K1—C1—N1—C3	−114.58 (16)	C7—C6—N2—K1	−15.8 (2)
K1^i^—C1—N1—C3	172.45 (19)	C8—C6—N2—K1	−136.00 (15)
N2—C1—N1—K1^i^	8.79 (17)	N4—C2—N3—C1	4.0 (3)
N3—C1—N1—K1^i^	−173.54 (12)	N4—C2—N3—C9	178.04 (16)
K1—C1—N1—K1^i^	72.97 (4)	N2—C1—N3—C2	88.5 (2)
N2—C1—N1—K1	−64.18 (16)	N1—C1—N3—C2	−89.2 (2)
N3—C1—N1—K1	113.48 (14)	K1—C1—N3—C2	−3.3 (2)
K1^i^—C1—N1—K1	−72.97 (4)	N2—C1—N3—C9	−85.4 (2)
C4—C3—N1—C1	113.8 (2)	N1—C1—N3—C9	97.0 (2)
C5—C3—N1—C1	−125.92 (19)	K1—C1—N3—C9	−177.14 (12)
C4—C3—N1—K1^i^	−78.5 (2)	C11—C9—N3—C2	−171.40 (18)
C5—C3—N1—K1^i^	41.8 (3)	C10—C9—N3—C2	65.6 (2)
C4—C3—N1—K1	18.8 (2)	C11—C9—N3—C1	2.4 (3)
C5—C3—N1—K1	139.15 (14)	C10—C9—N3—C1	−120.61 (19)
N1—C1—N2—C6	176.79 (17)	N3—C2—N4—C12	176.46 (17)
N3—C1—N2—C6	−0.9 (3)	N3—C2—N4—K1	−2.6 (2)
K1—C1—N2—C6	114.86 (16)	C14—C12—N4—C2	129.07 (19)
K1^i^—C1—N2—C6	−174.49 (19)	C13—C12—N4—C2	−109.7 (2)
N1—C1—N2—K1^i^	−8.72 (17)	C14—C12—N4—K1	−51.9 (2)
N3—C1—N2—K1^i^	173.63 (13)	C13—C12—N4—K1	69.4 (2)
K1—C1—N2—K1^i^	−70.65 (4)	C16^ii^—C16—O1—C15	−172.2 (3)
N1—C1—N2—K1	61.93 (15)	C16^ii^—C16—O1—K1	66.3 (3)
N3—C1—N2—K1	−115.72 (14)	K1—C15—O1—C16	−131.5 (2)
K1^i^—C1—N2—K1	70.65 (4)		

Symmetry codes: (i) −*x*, −*y*, −*z*+2; (ii) −*x*, −*y*−1, −*z*+2.
